# An unusually high production of hepatic aflatoxin B_1_-dihydrodiol, the possible explanation for the high susceptibility of ducks to aflatoxin B_1_

**DOI:** 10.1038/s41598-019-44515-6

**Published:** 2019-05-29

**Authors:** Gonzalo J. Diaz, Hansen W. Murcia

**Affiliations:** Laboratorio de Toxicología, Facultad de Medicina Veterinaria y de Zootecnia, Universidad Nacional de Colombia, Bogotá, D.C. 111321 Colombia

**Keywords:** Oxidoreductases, Multienzyme complexes

## Abstract

A study was conducted to determine the enzymatic kinetic parameters V_*max*_, K_*M*_, and intrinsic clearance (CL_*int*_) for the hepatic *in vitro* production of aflatoxin B_1_-dihydrodiol (AFB_1_-dhd) from aflatoxin B_1_ (AFB_1_) in four commercial poultry species, ranging in sensitivity to AFB_1_ from highest (ducks) to lowest (chickens). Significant but small differences were seen for V_*max*_, while large significant differences were observed for K_*M*_. However, the largest inter-species differences were observed for the CL_*int*_ parameter, with ducks being extraordinarily efficient in converting AFB_1_ into AFB_1_-dhd. Since AFB_1_-dhd is considered the metabolite responsible for the acute toxic effects of AFB_1_, the high hepatic production of AFB_1_-dhd from AFB_1_ in ducks is the possible biochemical explanation for the extraordinary high sensitivity of this poultry species to the adverse effects of AFB_1_.

## Introduction

Since the discovery of aflatoxins^1^ after the turkey “X” disease outbreak that killed over 100,000 turkey poults in Britain in 1960^2^, it has been known that there are extremely large differences in sensitivity to aflatoxin B_1_ (AFB_1_) among commercial poultry species. The sensitivity to the acute effects of AFB_1_, expressed as LD_50_ values, ranges from 0,4 mg/kg in day-old ducklings^3^ to 6,8 mg/kg in day-old chicks^4^. The minimum dietary concentration of AFB_1_ capable of affecting growth in ducks, turkeys, chickens and laying hens is about 50, 200, 500 and 5000 ng/kg when exposed to the toxic diets for 3 to 4 weeks^5^. The 100-fold difference between ducks and laying hens reflects the extreme tolerance to aflatoxins in adult chickens and the large sensitivity in ducks. Chickens even grow better when there are aflatoxins in their diet^6^, whereas ducks are so sensitive that they were used as a biological assay for testing feedstuffs^7^, prior to the development of the modern analytical techniques for aflatoxins.

**Figure 1:**
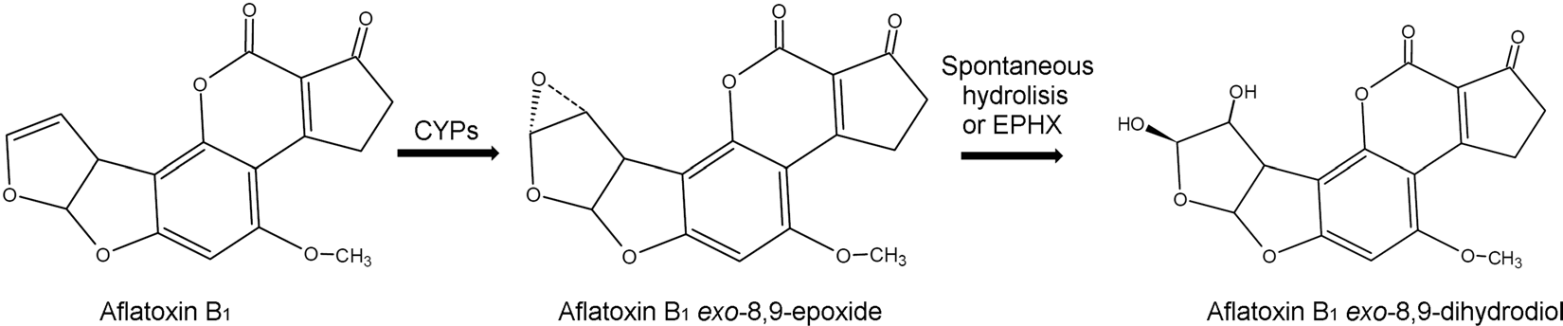
Biotransformation route of aflatoxin B_1_ into aflatoxin B_1_ *exo*-8,9-dihydrodiol. CYPs: Cytochrome P450. EPHX: Epoxide hydrolase.

To become a toxic compound, AFB_1_ requires biotransformation by cytochrome P450 enzymes (CYP). Several AFB_1_ metabolites from mammalian and avian CYPs have been identified including aflatoxins M_1_, B_2*a*_, P_1_ and Q_1_, and the electrophilic unstable AFB_1_-*exo*-8,9-epoxide (AFBO)^8^. The epoxide can alkylate RNA *in vitro*^9^ as well as the N7 position of guanine residues in DNA, forming irreversible adducts^10^; these adducts eventually cause the transversion G → T at codon 249 of the p53 tumor suppressor gene in human hepatocytes^11^, leading to hepatic cancer. Chronic exposure to AFB_1_ causes hepatocellular carcinoma not only in humans but also in such species as rats, primates and ducks^8^. The AFB_1_ metabolite responsible for the acute toxic effects of AFB_1_ has not been clearly identified but one possible candidate is the AFB_1_-*exo*-8,9-dihydrodiol (AFB_1_-dhd) that results from the nucleophilic trapping process of the AFB_1_-*exo*-8,9-epoxide by water^12,13^, Fig. 1. AFB_1_-dhd has been shown to inhibit protein synthesis *in vitro*^14^ and its furofuran-ring-opened oxyanionic metabolite (AFB_1_-hydroxydialdehyde) can form lysine adducts in serum albumin *in vivo*^15,16^. Further, an aldehyde reductase with activity toward the AFB_1_ dialdehyde has been associated with decreased liver toxic effects in rats^17^. Therefore, the dihydrodiol/dialdehyde forms, which occur in equilibrium at physiological pH^17^, appear to be responsible for the cytotoxic acute effects of AFB_1_ exposure. For more than a decade our research group has been looking for biochemical differences in the hepatic biotransformation of AFB_1_ that could explain the *in vivo* differences in response to AFB_1_ among the main poultry species^8,18–21^. The present study shows for the first-time large differences in the enzymatic kinetic parameters of AFB_1_-dhd production in liver microsomes that could explain the different *in vivo* sensitivity to AFB_1_ of resistant (chickens and quail), sensitive (turkeys) and highly sensitive (ducks) poultry species.

## Results

**Figure 2:**
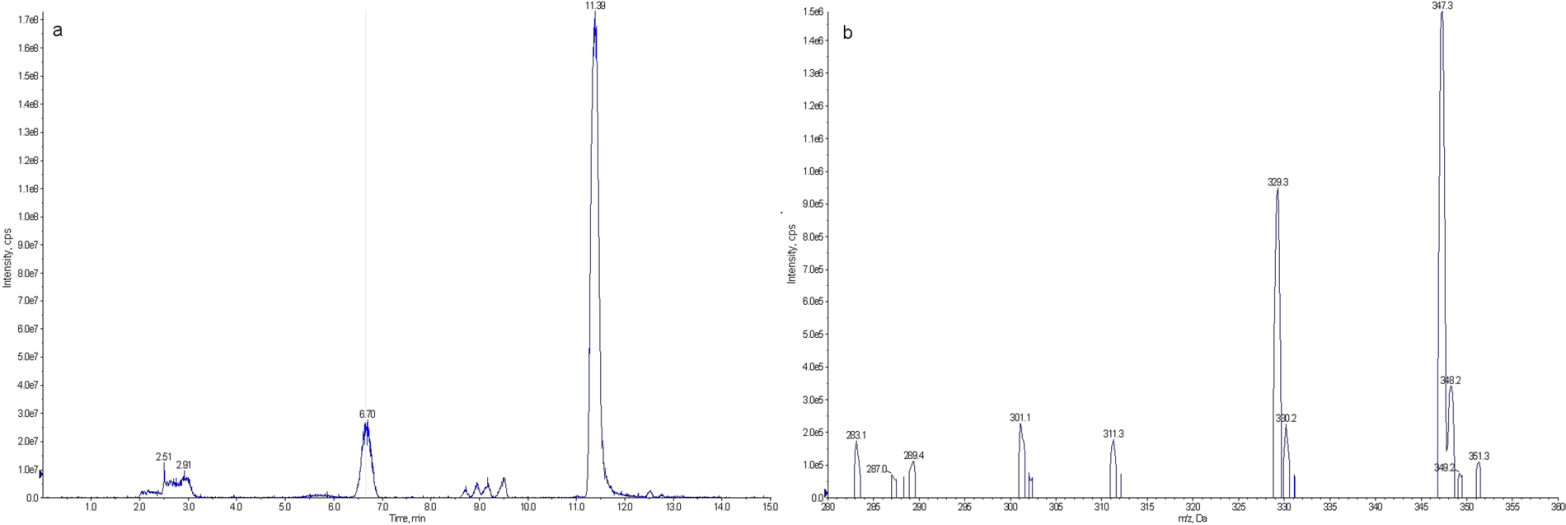
Identification of AFB_1_-dhd by HPLC-MS. (**a**) Chromatogram of a microsomal incubation showing the putative AFB_1_-dhd peak (*t*_R_ = 6.70) and AFB_1_ (*t*_R_ = 11.38). (**b**) Protonated monoisotopic masses found in the 6.70 min peak: the mass of 347.3 Da corresponds to the mass of AFB_1_-dhd whereas the mass of 329.3 Da (−18 Da) most likely corresponds to the dehydrated form of AFB_1_-dhd.

Due to the lack of a commercially available AFB_1_-dhd standard, a mass spectrometric analysis of the putative AFB_1_-dhd peak was conducted to determine its monoisotopic mass. The putative peak observed at 6.7 min Fig. 2a corresponded to a compound of 347 Da, which is consistent with the monoisotopic protonated mass of AFB_1_-dhd Fig. 2b.

**Figure 3:**
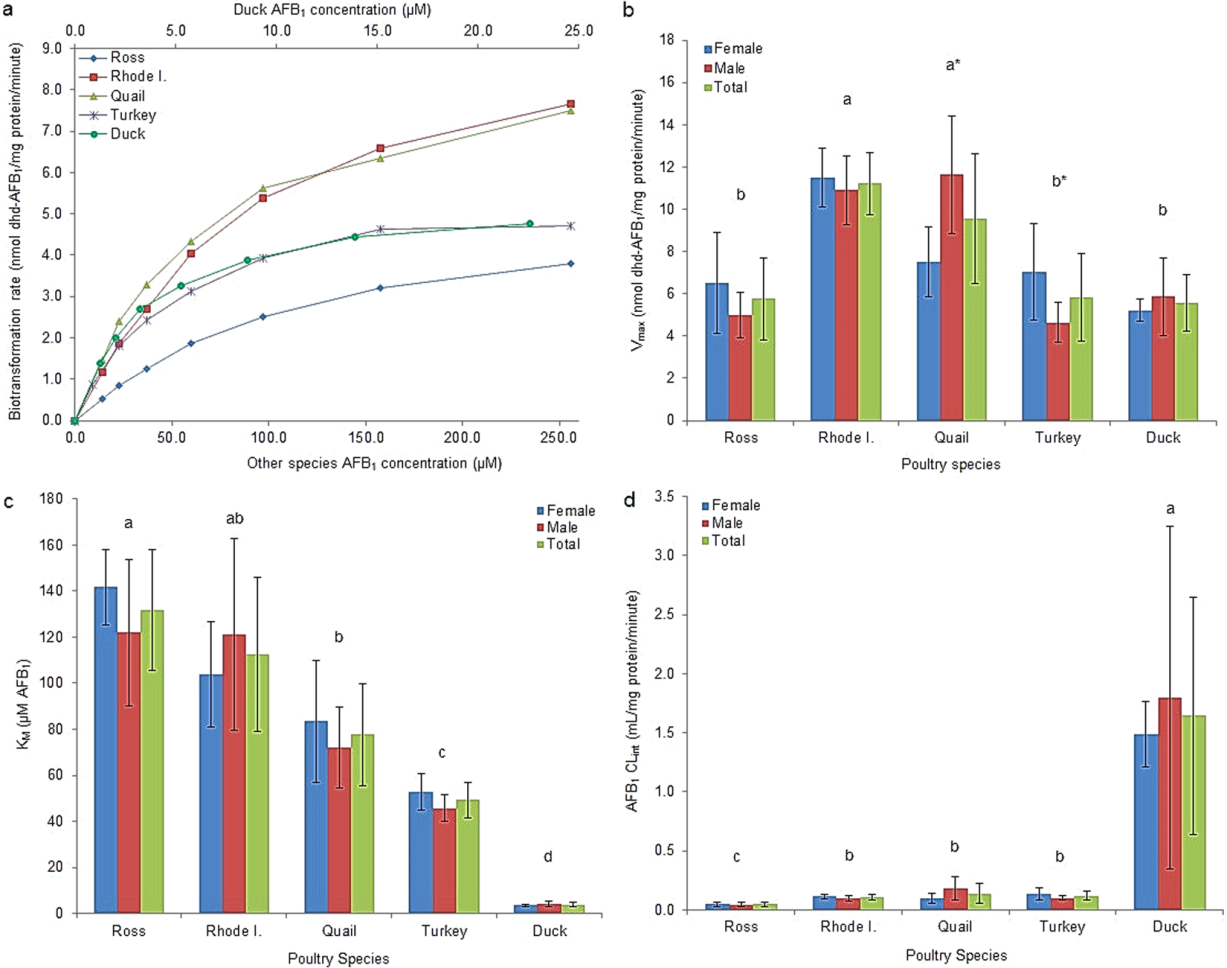
Enzyme kinetic parameters of microsomal *in vitro* AFB_1_-dhd production. (**a**) Saturation curve at AFB_1_ concentrations of 13.9 to 256 *μ*M (incubations with duck microsomal fraction were done at AFB_1_ concentrations of 1.23 to 22.6 *μ*M). (**b**) Maximal velocity (V_*max*_). (**c**) Michaelis-Menten constant (K_*M*_). (**d**) Intrinsic clearance (CL_*int*_; V_*max*_/K_*M*_). Total mean values with the same letter do not differ significantly. (*) Statistical differences by sex (P < 0.05) were calculated by the Kruskal-Wallis test.

The enzymatic kinetic parameters for AFB_1_-dhd production by the four poultry species investigated are presented in Fig. 3. Chicken and quail enzymes did not saturate even at the highest AFB_1_ concentration evaluated (256 *μ*M) Fig. 3a; however, turkey and duck enzymes seemed to become completely saturated with only 56 *μ*M AFB_1_. The average values for the V_*max*_ were the highest in Rhode Island Red chickens (11.2 ± 1.48 nmol of dhd-AFB_1_/mg protein/minute) and quail (9.57 ± 3.06 nmol of dhd-AFB_1_/mg protein/minute), while no differences (P > 0.05) were observed among Ross chickens, turkeys and ducks (5.75 ± 1.95, 5.84 ± 2.07 and 5.55 ± 1.33 nmol of dhd-AFB_1_/mg protein/minute, respectively) Fig. 3b. Rhode Island Red chicks had a higher V_*max*_ value compared with Ross chickens. Regarding differences by sex, only quail and turkeys showed significant differences between males and females. The average values for K_*M*_ showed large (P < 0.05) differences, with ducks presenting the lowest K_*M*_ value by far (3.84 ± 1.01 *μ*M of AFB_1_), followed by turkeys (49.33 ± 7.66 *μ*M of AFB_1_), quail (77.79 ± 22.14 *μ*M of AFB_1_) and the chicken breeds Rhode Island Red and Ross (112.5 ± 33.4 and 131.8 ± 26.2 *μ*M of AFB_1_, respectively) Fig. 3c. No differences between males and females were found in any species for this enzyme kinetic parameter. Further, no differences between the chicken breeds were found either. Regarding the CL_*int*_ parameter, very large differences among the species evaluated were observed, with ducks being extraordinarily efficient in converting AFB_1_ into AFB_1_-dhd compared to the other poultry species investigated Fig. 3d. CL_*int*_ values for ducks, turkey, quail and Rhode Island Red and Ross chickens were 1.64 ± 1.00, 0.12 ± 0.04, 0.14 ± 0.08, 0.11 ± 0.02 and 0.05 ± 0.02 mL/mg protein/minute, respectively. No differences between males and females were observed.

## Discussion

Since the discovery of aflatoxins in the early 1960’s it was observed that different animal species exhibit very different adverse effects upon exposure to the toxins. For example, ducklings, pigs and dogs die acutely at dietary concentrations that are well tolerated by humans, chickens and rats^22–24^. In some animal models, these differences can be explained through a differential hepatic biotransformation of AFB_1_. For instance, in mice and rats, differences in the ability to trap AFB_1_ with glutathione (GSH) ultimately determine the degree of AFB_1_-induced liver damage: while rats develop hepatocellular carcinoma upon chronic exposure to AFB_1_, mice are resistant. The reason for this differential response lies in the constitutive expression of high levels of an Alpha-class glutathione transferase (GST) that catalyzes the trapping of AFBO in the mouse that is only expressed at low levels in the rat^25^. Among poultry species exposed chronically to AFB_1_ the only one that develops liver cancer is the duck^26^; however, due to the short life-span of commercial poultry, it is actually the acute effects the ones that are more important. For more than a decade our research group has been searching for a biochemical explanation for the differences in susceptibility to AFB_1_ among the main poultry species. We have found that AFB_1_ is essentially biotransformed into aflatoxicol and AFB_1_-dhd by chicken, quail, turkey and duck liver microsomes and that at least four CYPs can bioactivate AFB_1_ into the epoxide in ducks, whereas CYP2A6 is the main cytochrome responsible for this reaction in chickens, quail and turkeys^8,18–21^. However, none of these findings could explain the extraordinarily high sensitivity of the duck compared to other poultry.

In the present study we investigated the *in vitro* kinetic constants V_*max*_ and K_*M*_, as well as their ratio, also known as intrinsic clearance. Measurement of CL_*int*_ has been used to predict the hepatic extraction of a compound^27^, and it is considered to be a measure of the total amount of enzyme that is coupled to the substrate and engaged in the conversion of the substrate into the product^28^, in other words it is a means to express enzyme efficiency^29^. Maximal velocity did not differ significantly between duck and turkey (sensitive species) or Ross chickens (highly resistant species); however, large significant differences in K_*M*_ were seen among the poultry species studied. Duck presented the lowest value: almost 13 times lower that turkey, 20 times lower than quail and 30 times lower than the chicken breeds. The calculation of the CL_*int*_ values revealed that duck liver microsomes clear AFB_1_ as AFB_1_-dhd at rates between 15 and 33 times higher than chickens. These values are due the low duck K_*M*_ values for AFB_1_-dhd production, which means that duck CYPs require very low concentrations of AFB_1_ to reach maximal velocity. More tolerant or resistant species require higher amounts of AFB_1_ to reach V_*max*_, making their CYP enzymes a low performance biotransformation system. Based on these results we propose an order of AFB_1_ clearance as AFB_1_-dhd in the poultry species studied as follows: duck ⋙ quail > turkey > chicken (Ross), with values of 1.64, 0.14, 0.12 and 0.05 mL/mg/minute, respectively. In regard to differences between males and females we confirmed previous results obtained in our laboratory, where no significant differences were found by sex.

In summary, the present findings not only provide a biochemical explanation for the large differences in susceptibility to AFB_1_ between chickens and ducks, but also provide strong evidence that AFB_1_-dhd is the metabolite responsible for the acute toxicity of AFB_1_. We hypothesize that the large production of AFB_1_-dhd by the duck liver is the cause of the mortality and liver lesions observed with dietary concentrations that do not affect other poultry. Further, the large production of AFB_1_-dhd, which is in turn produced by the AFB_1_-*exo*-8,9-epoxide, might be related to the fact that ducks are the only poultry species that develop hepatic cancer after AFB_1_ exposure.

## Methods

### Reagents

AFB_2*a*_, glucose 6-phosphate sodium salt, glucose 6-phosphate dehydrogenase, nicotinamide dinucleotide phosphate (NADP^+^), ethylenediaminetetraacetic acid (EDTA), bicinchoninic acid solution (sodium carbonate, sodium tartrate, sodium bicarbonate and sodium hydroxide 0.1 N pH 11.25), copper sulphate pentahydrate, formic acid, dimethylsulfoxide (DMSO), sucrose, glycerol, and bovine serum albumin were from Sigma-Aldrich (St. Louis, MO, USA). Aflatoxin B_1_ was from Fermentek Ltd. (Jerusalem, Israel). Sodium chloride and magnesium chloride pentahydrate were purchased from Mallinckrodt Baker (Phillipsburg, NJ, USA). Sodium phosphate monobasic monohydrate and sodium phosphate dibasic anhydrous were from Merck (Darmstadt, Germany). Methanol, acetonitrile and water were all HPLC grade.

### Microsomal fraction processing

Liver fractions were obtained from 12 healthy birds (6 males and 6 females) from each of the following species and age: seven-week old Ross and Rhode Island Red chickens (*Gallus gallus ssp. domesticus*), eight-week old turkeys (*Meleagris gallopavo*), eight-week old quails (*Coturnix coturnix japonica*) and nine-week old Pekin ducks (*Anas platyrhynchos ssp. domesticus*). The birds were sacrificed by cervical dislocation, and their livers extracted immediately, washed with cold PBS buffer (50 mM phosphates, pH 7.4, NaCl 150 mM), cut into small pieces and stored at −70 °C until processing. The experiment was conducted following the welfare guidelines of the Poultry Research Facility and was approved by the Bioethics Committee, Faculty of Veterinary Medicine and Zootechnics, National University of Colombia, Bogotá D.C., Colombia (approval document CB-FMVZ-UN-033-18). Frozen liver samples were allowed to thaw, and 2.5 g were minced and homogenized for 1 minute with a tissue homogenizer (Cat X120, Cat Scientific Inc., Paso Robles, CA, USA) with 10 mL of extraction buffer (phosphates 50 mM pH 7.4, EDTA 1 mM, sucrose 250 mM). The homogenates were then centrifuged at 12,000 × *g* for 30 minutes at 4 °C (IEC CL31R Multispeed Centrifuge, Thermo Scientific, Waltham, MA, USA). After this first centrifugation, the supernatants (approximately 10 mL) were transferred into ultracentrifuge tubes kept at 4 °C and centrifuged for 90 minutes at 100,000 × *g* (Sorval WX Ultra 100 Centrifuge, Thermo Scientific, Waltham, MA, USA). The resulting pellets (corresponding to the microsomal fraction) were resuspended in 3 mL of storage buffer (phosphates 50 mM pH 7.4, EDTA 1 mM, sucrose 250 mM, 20% glycerol), fractioned in microcentrifuge tubes and stored at −70 °C. An aliquot of each sample was taken to determine its protein content by using the bicinchoninic acid protein quantification method according to Redinbaugh and Turley^30^.

### Microsomal incubations

Incubations were carried out in 1.5 mL microcentrifuge tubes kept at 39 °C (the normal average avian body temperature) containing 5 mM glucose 6-phosphate, 0.5 I.U. of glucose 6-phosphate dehydrogenase, 0.5 mM NADP^+^, 1 *μ*L of AFB_1_ in DMSO at concentrations ranging from 1.23 to 256 *μ*M, and 5 *μ*g of microsomal protein. All volumes were completed with incubation buffer (phosphates 50 mM pH 7.4, MgCl 5 mM, EDTA 0.5 mM), and the reaction stopped after 10 minutes with 250 *μ*L of ice-cold acetonitrile. The stopped incubations were centrifuged at 15,000 × *g* for 10 minutes and 2 *μ*L of a 1:10 dilution in mobile phase were analyzed by High Performance Liquid Chromatography (HPLC) as described below.

### Chromatographic conditions (HPLC)

The production of AFB_1_-dhd in each incubation was quantitated in a Shimadzu Prominence system (Shimadzu Scientific Instruments, Columbia, MD, USA) equipped with a DGU-20A3R degassing unit, two LC-20AD pumps, a SIL-20AC_*HT*_ autosampler, a CTO-20A column oven, an SPD-20AV UV-Vis detector, an RF-20A_*XS*_ fluorescence detector, and a CBM-20A bus module, all controlled by “LC Solutions” software. The chromatography was carried out on an Alltech Alltima HP C18, 150 mm × 3.0 mm (Alltech Associates Inc., Deerfield, IL, USA) kept at 40 °C. The mobile phase was a linear gradient of solvent A (water − 0.1% formic acid) and B (acetonitrile:methanol, 1:1–0.1% formic acid), as follows: 0 min: 25% B, 1 min: 25% B, 10 min: 60% B, 10.01 min: 25% B, and 17 min: 25% B. The flow rate was 0.4 mL/min and the fluorescence detector was set at excitation and emission wavelengths of 365 nm and 425 nm, respectively. The in-vial concentration of AFB_1_-dhd was quantitated using an external standard of AFB_2*a*_, since these two compounds share identical spectral properties^9^. Further, the monoisotopic protonated mass of AFB_1_-dhd was determined by HPLC-MS by means of a 3200 QTrap mass spectrometer (Applied Biosystems, Toronto, Canada) using a thermospray ionization probe in positive mode and the following settings: probe voltage: 4,800 V, declustering potential: 140 V, entrance potential: 10 V, curtain gas value: 30, collision energy: 81 V and collision cell exit potential: 5 V.

### Statistical analysis

The enzymatic parameters K_*M*_ and V_*max*_ were determined by non-linear regression using the Marquardt method adjusting the data to the Michaelis-Menten enzyme kinetics using the equation: v = V_*max*_[S]/K_*M*_ + [S], where v is the enzyme reaction velocity, [S] represents substrate concentration, V_*max*_ represents maximal velocity and K_*M*_ represents the Michaelis-Menten constant. Intrinsic clearance (CL_*int*_) was calculated as the ratio V_*max*_/K_*M*_. Inter-species differences in enzymatic kinetic parameters were determined by using the Kruskal-Wallis test, while nonparametric multiple comparisons were made by using the Dwass-Steel-Critchlow-Fligner method. All analyses were performed using the Statistical Analysis System software^31^.

## Data Availability

The datasets generated during and/or analysed during the current study are available from the corresponding author on reasonable request.
